# National health examination surveys; a source of critical data

**DOI:** 10.2471/BLT.24.291783

**Published:** 2024-06-04

**Authors:** Paula Margozzini, Hanna Tolonen, Antonio Bernabe-Ortiz, Sarah Cuschieri, Chiara Donfrancesco, Luigi Palmieri, Luz Maria Sanchez-Romero, Jennifer S Mindell, Oyinlola Oyebode

**Affiliations:** a Department of Public Health, Pontificia Universidad Católica de Chile, Santiago, Chile.; b Finnish Institute for Health and Welfare, Helsinki, Finland.; c Universidad Científica del Sur, Lima, Peru.; d Faculty of Medicine and Surgery, University of Malta, Msida, Malta.; e Istituto Superiore di Sanità, Rome, Italy.; f Department of Oncology, Georgetown University Medical Centre, Washington DC., United States of America.; g Department of Epidemiology and Public Health, University College London, 1–19 Torrington Place, London, WC1E 6BT, England.; h Wolfson Institute of Population Health, Queen Mary University of London, London, England.

## Abstract

The aim of this paper is to contribute technical arguments to the debate about the importance of health examination surveys and their continued use during the post-pandemic health financing crisis, and in the context of a technological innovation boom that offers new ways of collecting and analysing individual health data (e.g. artificial intelligence). Technical considerations demonstrate that health examination surveys make an irreplaceable contribution to the local availability of primary health data that can be used in a range of further studies (e.g. normative, burden-of-disease, care cascade, cost and policy impact studies) essential for informing several phases of the health planning cycle (e.g. surveillance, prioritization, resource mobilization and policy development). Examples of the use of health examination survey data in the World Health Organization (WHO) European Region (i.e. Finland, Italy, Malta and the United Kingdom of Great Britain and Northern Ireland) and the WHO Region of the Americas (i.e. Chile, Mexico, Peru and the United States of America) are presented, and reasons why health provider-led data cannot replace health examination survey data are discussed (e.g. underestimation of morbidity and susceptibility to bias). In addition, the importance of having nationally representative random samples of the general population is highlighted and we argue that health examination surveys make a critical contribution to external quality control for a country’s health system by increasing the transparency and accountability of health spending. Finally, we consider future technological advances that can improve survey fieldwork and suggest ways of ensuring health examination surveys are sustainable in low-resource settings.

## Résumé

Cet article a pour objet d’apporter des arguments techniques au débat sur l’importance des enquêtes de santé par examen et sur leur utilisation continue pendant la crise post-pandémique du financement de la santé et dans le contexte d’un essor de l’innovation technologique qui offre de nouvelles façons de collecter et d’analyser les données individuelles sur la santé (comme l’intelligence artificielle). Les considérations techniques démontrent que les enquêtes de santé par examen apportent une contribution irremplaçable à la disponibilité locale de données de santé primaires qui peuvent servir dans une série d’études complémentaires (telles que des études normatives, sur la charge de morbidité, la cascade des soins, les coûts et l’impact des politiques). Ces études sont essentielles pour renseigner plusieurs phases du cycle de planification sanitaire (par exemple: surveillance, priorisation, mobilisation de ressources et élaboration de politiques). Cet article présente des exemples d’utilisation des données d’enquêtes de santé par examen dans la Région OMS de l’Europe (Finlande, Italie, Malte et Royaume-Uni de Grande-Bretagne et d’Irlande du Nord) et dans la Région OMS des Amériques (Chili, États-Unis d’Amérique, Mexique et Pérou) et aborde les raisons pour lesquelles les données fournies par les prestataires de soins de santé ne peuvent pas remplacer les données d’enquêtes de santé par examen (par exemple la sous-estimation de la morbidité et la vulnérabilité aux biais). En outre, il soulignet l’importance de disposer d’échantillons aléatoires représentatifs de la population générale au niveau national, et nous soutenons que les enquêtes de santé par examen apportent une contribution essentielle au contrôle externe de la qualité du système de santé d’un pays en renforçant la transparence des dépenses de santé et l’obligation de rendre des comptes à leur sujet. Enfin, nous envisageons les futures avancées technologiques susceptibles d’améliorer le travail d’enquête sur le terrain et suggérons des moyens d’assurer la viabilité des enquêtes de santé par examen dans les environnements à faibles ressources.

## Resumen

El objetivo de este artículo es aportar argumentos técnicos al debate sobre la importancia de las encuestas de salud y su uso continuado durante la crisis de financiación sanitaria pospandémica y en el contexto de un auge de la innovación tecnológica que ofrece nuevas formas de recopilar y analizar datos sanitarios individuales (por ejemplo, la inteligencia artificial). Las consideraciones técnicas demuestran que las encuestas de salud contribuyen de manera insustituible a la disponibilidad local de datos sanitarios primarios que pueden utilizarse en toda una serie de estudios posteriores (por ejemplo, estudios normativos, de carga de morbilidad, de cascada asistencial, de costes y de impacto de las políticas) esenciales para fundamentar varias fases del ciclo de planificación sanitaria (por ejemplo, vigilancia, establecimiento de prioridades, movilización de recursos y elaboración de políticas). Se presentan ejemplos del uso de los datos de las encuestas de salud en la Región Europea de la Organización Mundial de la Salud (Finlandia, Italia, Malta y el Reino Unido de Gran Bretaña e Irlanda del Norte) y en la Región de las Américas de la OMS (Chile, Estados Unidos de América, México y Perú) y se analizan las razones por las que los datos obtenidos por los proveedores sanitarios no pueden sustituir a los de las encuestas de salud (por ejemplo, la subestimación de la morbilidad y la posibilidad de sesgo). Además, se destaca la importancia de contar con muestras aleatorias representativas de la población general a escala nacional y se argumenta que las encuestas de salud contribuyen de forma decisiva al control de calidad externo del sistema sanitario de un país, al aumentar la transparencia y la rendición de cuentas del gasto sanitario. Por último, se examinan los futuros avances tecnológicos que pueden mejorar el trabajo de campo de las encuestas y se sugieren métodos para garantizar que las encuestas de salud sean sostenibles en entornos con pocos recursos.

## ملخص

الهدف من هذه الورقة هو الإسهام بالنقاشات التقنية في الجدال حول أهمية مسوحات الفحص الصحي، واستخدامها المتواصل خلال أزمة التمويل الصحي فيما بعد الجائحة، وفي سياق طفرة الابتكار التكنولوجي التي تقدم طرقًا جديدة لجمع البيانات الصحية للأفراد وتحليلها (مثل الذكاء الاصطناعي). توضح الاعتبارات التقنية أن مسوحات الفحص الصحي تقدم إسهامًا لا غنى عنه في التوفر المحلي للبيانات الصحية الأولية التي يمكن استخدامها في مجموعة من الدراسات الإضافية (مثل الدراسات المعيارية، ودراسات عبء المرض، ودراسات سلسلة الرعاية، ودراسات التكاليف وتأثير السياسات)، والتي تعد دراسات ضرورية لإرشاد عدة مراحل من دورة التخطيط الصحي (مثل المراقبة، وتحديد الأولويات، وتعبئة الموارد، ووضع السياسات). تتضح أمثلة استخدام بيانات مسح الفحص الصحي في المنطقة الأوروبية لمنظمة الصحة العالمية (أي فنلندا وإيطاليا ومالطة والمملكة المتحدة لبريطانيا العظمى وأيرلندا الشمالية)، ومنطقة الأمريكتين لمنظمة الصحة العالمية (أي شيلي والمكسيك وبيرو والولايات المتحدة الأمريكية). كما يتم مناقشة الأسباب التي تجعل البيانات التي يقدمها مقدمو الخدمات الصحية لا يمكنها أن تحل محل بيانات مسح الفحص الصحي (على سبيل المثال، التقليل من قيمة معدلات الإصابة بالأمراض، والحساسية تجاه التحيز). بالإضافة إلى ذلك، تم التركيز على أهمية وجود عينات عشوائية تمثيلية على المستوى الوطني لعامة السكان، ونحن نزعم أن مسوحات الفحص الصحي تقدم إسهامًا حيويًا في مراقبة الجودة الخارجية للنظام الصحي للدولة من خلال زيادة مستويات الشفافية وقابلية المساءلة في قطاعات الإنفاق على الصحة. وأخيراً، فإننا نضع في الاعتبار التقدم التكنولوجي المستقبلي الذي من شأنه أن يُحسّن العمل الميداني للمسح، ويقترح طرقًا لضمان استدامة مسوحات الفحص الصحي في البيئات ذات الموارد المنخفضة.

## 摘要

本文目的在于为关于健康检查调查的重要性及其在大流行后卫生融资危机期间和技术创新热潮背景下的持续使用的辩论提供技术论据。技术创新热潮为收集和分析个人健康数据提供了新方法（例如人工智能）。技术问题表明，健康检查调查对在当地获得基层卫生数据作出了不可替代的贡献，这些数据可用于一系列的进一步研究（例如规范性研究、疾病负担研究、护理级联研究、成本和政策影响研究），这些研究为卫生规划周期的几个阶段（例如监测、确定优先次序、资源调动和政策制定）提供了重要的依据。我们还列举在世界卫生组织欧洲区域（即大不列颠及北爱尔兰联合王国、芬兰、马耳他和意大利)和世卫组织美洲区域（即秘鲁、美利坚合众国、墨西哥和智利）使用健康检查调查数据的例子，并讨论了卫生服务提供者主导的数据不能取代健康检查调查数据的原因（例如低估发病率和偏倚易感性）。此外，还强调了对一般人群进行具有全国代表性的随机抽样的重要性，我们认为，健康检查调查通过提高卫生服务支出的透明度和改进相关问责制，对一个国家卫生服务系统的外部质量控制做出了重要贡献。最后，我们考虑了未来的技术进步，这些技术进步有助于改善调查实地工作，并提出确保健康检查调查在资源匮乏环境中可合理利用的方法。

## Резюме

Цель данной статьи заключается в том, чтобы внести технические аргументы в дискуссию о важности проведения опросов по изучению состояния здоровья и их дальнейшем использовании в период постпандемического кризиса финансирования здравоохранения и в контексте расцвета технологических инноваций, предлагающих новые способы сбора и анализа индивидуальных данных о здоровье (например, искусственный интеллект). Технические особенности свидетельствуют о том, что результаты опросов по изучению состояния здоровья вносят незаменимый вклад в обеспечение наличия на местном уровне первичных данных о состоянии здоровья, которые могут использоваться в целом ряде дальнейших исследований (например, нормативные исследования, исследования бремени болезней, каскада ухода, затрат и влияния на политику), необходимых для информационного обеспечения нескольких этапов цикла планирования здравоохранения (например, наблюдения, определения приоритетов, мобилизации ресурсов и разработки политики). Приведены примеры использования данных опросов по изучению состояния здоровья в Европейском регионе Всемирной организации здравоохранения (например, в Италии, Соединенном Королевстве Великобритании и Северной Ирландии, Финляндии и на Мальте) и Американском регионе ВОЗ (например, в Мексике, Перу, Соединенных Штатах Америки и Чили) и рассмотрены причины, по которым полученные от медицинских работников данные не могут заменить данные опросов по изучению состояния здоровья (например, недооценка заболеваемости и подверженность предвзятости). Кроме того, подчеркивается важность наличия национально репрезентативных случайных выборок населения в целом, на основании чего можно утверждать, что опросы по изучению состояния здоровья вносят важнейший вклад во внешний контроль качества системы здравоохранения страны, повышая прозрачность и подотчетность расходов на здравоохранение. В заключение рассматриваются будущие технологические достижения, которые могут улучшить работу на местном уровне, и предлагаются способы обеспечения устойчивости проведения опросов по изучению состояния здоровья в условиях малого количества ресурсов.

## Introduction

National health examination surveys have been developed to gather important information that cannot be obtained from other sources. In these surveys, trained field staff take objective, biophysical measurements (e.g. of anthropometric variables or blood pressure) and collect biological samples (e.g. of blood or urine) for laboratory analysis. The data obtained complement the self-reported data collected, for example, by health interview surveys, which include only self-reported information. In addition, health examination surveys are the observational studies with the greatest external validity because they are based on randomized, representative household samples. Consequently, the information obtained is relevant for both population (i.e. public health) and individual health.^1^

Health examination surveys provide more accurate information than health interview surveys. For example, people tend to overestimate their height and underestimate their weight compared with measurements taken by trained staff, which results in underestimates of their body mass index – a measure widely used for assessing people for obesity and for predicting morbidity from several chronic noncommunicable diseases.^2^^,^^3^

Most high- and middle-income countries conduct national health interview surveys that use questionnaires to collect basic information about the general population. For European Union member states, European health interview surveys are mandatory.^4^ Far fewer countries regularly conduct health examination surveys. Nevertheless, many low- and middle-income countries have conducted at least one small health examination survey in accordance with the World Health Organization (WHO) STEPwise approach to noncommunicable disease risk factor surveillance, known as STEPS.^5^ In addition, many low- and middle-income countries conduct Demographic and Health Surveys (DHS), which are funded by the United States Agency for International Development, at least every 5 years and some (e.g. Peru) conduct them annually. These surveys include a small number of biophysical assessments, such as anthropometry and anaemia testing.^6^ However, few countries have a track record of frequent health examination surveys that include a wide range of biophysical measurements. The longest running health examination survey series in Europe has been conducted by Finland every 5 years since 1972 (i.e. the national FINRISK study).^7^ In the United States of America, National Health and Nutrition Examination Surveys started in the 1960s and have been run as a continuous programme since 1999.^8^

Existing national health examination surveys differ in the age groups covered, the range of measurements taken and the way they are organized. For example, most health examination surveys in mainland Europe and the United States make use of clinical examination centres, whereas those in the United Kingdom of Great Britain and Northern Ireland and Latin America involve visiting participants in their own homes.^9^^,^^10^ Despite some differences, they share many features, particularly sampling methods, survey questions, anthropometric measurements and some other biophysical measurement protocols.^1^^,^^9^^,^^10^

Although numerous reports and scientific papers have described findings based on health examination survey data, which implicitly suggests they are valuable data sources, few countries conduct regular, nationally representative, wide-ranging health examination surveys. Moreover, many countries that do conduct regular health examination surveys are struggling to secure long-term financing for successive surveys. Countries with small populations face additional challenges, including a lack of infrastructure and resources for conducting regular, nationally representative health examination surveys.^11^

Recently, several health examination surveys around the world have been delayed, survey field costs have risen and financing has been cut. In addition, advances in medical informatics and artificial intelligence have given rise to doubts about whether health examination surveys provide the most efficient means of collecting data. Apart from a few studies from Chile, England and Finland,^12^^–^^15^ little has been published on the usefulness of health examination surveys, their impact or factors influencing their effectiveness. In this context, it is possible to ask: (i) whether random general population samples are still needed for chronic disease surveillance and health planning; (ii) whether governments should still finance costly population health surveys; and (iii) whether new information technology could be used to generate chronic disease indicators that replace the field measurements conducted in health examination surveys.

The aim of this paper was to discuss these questions with reference to technical arguments and to examples drawn from health examination surveys conducted in Chile, England, Finland, Italy, Malta, Mexico, Peru and the United States. 

## Noncommunicable disease

Health examination surveys can provide primary data for use in different phases of the noncommunicable disease health planning cycle, including surveillance, prioritization, resource mobilization and policy formulation, implementation and impact assessment.

Table 1 shows some traditional noncommunicable disease indicators that can be obtained directly from health examination survey measures, and describes the specific contribution each of these indicators can make to deriving estimates of variables in other studies, such as: (i) studies to establish population norms; (ii) studies of the care cascade and health system performance; (iii) studies of health inequalities; (iv) studies of the burden of disease and of the burden attributable to specific risk factors; (v) studies of health costs and avoidable health costs; and (vi) studies of changes in population trends. Health policies and laws usually cite these studies directly but do not cite health examination surveys, which may hide their importance. In practice, national ministries of health could justify their budgets for health examination surveys by highlighting these contributions, and by giving examples of how health examination survey data can be used in local policy formulation, implementation and assessment. In Chile, health examination survey data have been used to supplement the findings of all the different study types listed in Table 1.

**Table 1:** Contribution of national health examination survey data to other study types essential for noncommunicable disease health planning

Study type	Data contributed by national health examination surveys to other study types					
	Health examination survey indicator					
	Risk factor prevalence	Self-reported diagnosis	Objective disease prevalence	Self-reported treatment and drug use	Biophysical parameters and disease control status	Perceived health and quality of life
Normative studies	General population and percentile age and sex risk factor exposure curves	National benchmarks for diagnostic coverage	National denominators for prevalence calculations and age- and sex-adjusted prevalence estimates for local administrative divisions	National benchmarks for treatment coverage	Population distribution curves and normal cut-offs for biophysical parameters; and national benchmarks for effective disease control coverage	National quality of life benchmarks; and local indexing of quality-of-life instruments
Care cascade and health system performance studies	NA	Diagnostic coverage	NA	Treatment coverage and inappropriate drug use	Effective treatment coverage	General population quality of life and disease-specific quality of life
Inequality studies	Estimates of relative and absolute excess risk factor exposure in underserved population groups	Gaps in diagnostic coverage in underserved population groups	Unequal disease distribution	Gaps in treatment coverage in underserved population groups	Gaps in effective treatment coverage in underserved population groups	Gaps in quality of life in underserved population groups
Studies of the global burden of disease and of the burden attributable to risk factors	Population attributable fractions and population impact fractions	NA	Prevalence-based estimates of the global burden of disease (DALYs and YLDs)	NA	NA	Estimates of country-specific disability weights
Health costs and avoidable costs studies	Attributable costs and avoidable costs due to changes in risk factor exposure (i.e. counterfactuals)	Avoidable costs attributable to increases in diagnostic coverage	Total burden-of-disease costs	Avoidable costs attributable to increases in treatment coverage	Avoidable costs attributable to improvements in treatment quality	Cost-effectiveness estimates (QALYs)
Changes in population trends	Changes in population trends in risk factor exposure	Changes in population trends in diagnostic coverage	Changes in population trends in disease prevalence and burden	Changes in population trends in treatment coverage	Changes in population trends in effective treatment coverage and biophysical parameter distributions	Changes in quality of life

DALY: disability-adjusted life-year; NA: not applicable; QALY: quality-adjusted life-year; YLD: year lived with disability.

Several noncommunicable disease preventive policies for nutrition, tobacco control and disease management (e.g. so-called WHO best buys) have been supported by, and their impact assessed, using health examination survey data. Examples of the influence of health examination surveys on policy in Chile, England, Finland, Italy, Malta, Mexico, Peru and the United States are described in Table 2 and Table 3.

**Table 2:** Examples of the influence of health examination surveys on dietary policy, Chile, England, Finland, Italy, Malta, Mexico, Peru and the United States, 1994–2020

Health topic and country	Stage at which health examination surveys influenced dietary policy^a^		
	Setting the agenda	Policy formation	Policy evaluation
**Obesity**			
Chile	The increases in obesity, an unhealthy diet and high urinary sodium levels reported in the 2010 *Encuesta Nacional de Salud* led to further studies of the local burden of disease and of the disease burden attributable to specific risk factors and to an obesity costs study^16^	These studies were decisive in establishing support for an increase in taxes on sugary beverages, front-of-package labelling for foods, the banning of unhealthy food sales in schools and the marketing regulations introduced in the 2014 food law. Based on *Encuesta Nacional de Salud* data,^16^ a new National Nutritional Policy was published in 2018, nutritional guidelines were updated and waist circumference cut-off points were defined for Chile^17^	NA
England, United Kingdom	General and abdominal obesity were monitored in adults and children using annual Health Survey for England data	The 2020 obesity strategy for England included: (i) a ban on television and online advertising of foods high in fat, sugar and salt before 21:00; (ii) restrictions on price promotions for these foods; (iii) restrictions on where shops may display these foods and unhealthy drinks; and (iv) a mandate for energy labelling of food in restaurants. These proposals were assessed during development of the obesity strategy using Health Survey for England anthropometric data in a Department of Health and Social Care calorie model that explored the likely impact of these and other possible policy interventions^18^	NA
Malta	Anthropometric data from health examination surveys were used to accurately estimate obesity^19^	These data helped in estimating the national cost of this disease and led to a range of stakeholders working together in a timely fashion to prevent obesity	NA
**Diet**			
Chile	Low serum folic acid and vitamin D levels and urinary iodine levels measured in the 2016–2017 *Encuesta Nacional de Salud*	In reaction to the observed ongoing nutritional transition, a national micronutrient strategy was developed in 2021 that updated requirements for the mandatory fortification of food^16^	Data from *Encuesta Nacional de Salud* surveys helped in monitoring the effect of changes in mandatory food fortification programmes^20^
Mexico	A DHS identified the double burden of malnutrition (i.e. undernutrition and overnutrition)^21^	NA	Health examination survey data demonstrated that social food assistance programmes reduced food insecurity;^22^ and DHSs were used to monitor the double burden of malnutrition and the effect of policies to address this burden^21^
**Vitamin D level**			
Finland	Data from the 2002 national FINRISK study showed low population levels of vitamin D^14^	These data supported the fortification of milk and fat spreads with vitamin D, which began in 2003^14^	Mean serum 25-hydroxyvitamin D levels rose from 48 nmol/L in 2000 to 65 nmol/L in 2011 (*P* < 0.001), with a higher rise of 20 nmol/L in survey participants not taking supplements^23^
**Salt intake**			
England, United Kingdom	Health examination survey data showed excessive sodium excretion, a marker for salt intake^24^	The Department for Health in England set targets in 2006 and worked with the food industry to reduce the salt content of the processed food products that contributed most to the population’s salt intake, as identified through health examination survey data (the initial focus was therefore on bread)	Significant falls in salt intake were observed from 2005–2006 to 2008–2009 but no further falls occurred by 2018–2019
Italy	24-hour urine samples taken in an health examination survey showed higher than recommended daily salt consumption in the adult population^25^	As a result, the health ministry, in collaboration with NGOs, undertook health promotion actions aimed at increasing awareness of the risks associated with excessive salt intake and reformulated a wide range of products to reduce their salt content in voluntary agreements with the food industry and artisan bakeries	24-hour urine samples showed a reduction in sodium excretion of around 12% between 2008–2012 and 2018–2019
**Nutritional status**			
Peru	A DHS identified high levels of maternal anaemia and childhood stunting	These findings informed the 2006 Children Malnutrition Initiative, which was accompanied by a National Strategy for Poverty Reduction and Economic Opportunities in 2007;^26^ and DHS data demonstrated the need to target social and nutritional programmes on rural areas^27^	These policies, alongside a cash transfer programme, were credited with the improvements seen in nutritional status^28^
United States	NA	National Health and Nutrition Examination Survey measurements provided the basis for the 2000 CDC growth charts for children, which updated the 1977 National Center for Health Statistics charts and created new body mass index-for-age charts^29^ – such population-level data can be used to identify outliers	NA
**Neural tube defects**			
United States	National Health and Nutrition Examination Survey data for 1988–1994 showed that the median red blood cell folate level in women aged 15–45 years was 160 ng/mL (substantially below the recommended 220 ng/mL); 38% of women had levels < 220 mg/mL^30^	In response, mandatory fortification of cereal grain products was implemented in January 1998	In the 1999–2000 National Health and Nutrition Examination Survey, the median red blood cell folate level in women aged 15–45 years was 264 ng/mL;^30^ the birth rate of babies born with neural tube defects fell in the 2 years before the mandatory fortification of cereal grain products, probably due to increased use of folate supplementation and prenatal diagnosis; the rate fell further in the 2 years after mandatory fortification; and, although the median folate level fell after 2000, the birth rate of babies born with neural tube defects continued to fall, though more slowly
**Lead intake**			
United States	National Health and Nutrition Examination Surveys have involved measuring population blood lead levels and a blood lead reference value is used to identify children who have blood lead levels above the 97.5th percentile for children aged 1–5 years^31^	Based on National Health and Nutrition Examination Survey data, federal policies and a range of public health interventions were introduced to reduce exposure to lead	National Health and Nutrition Examination Survey measurements of blood lead levels demonstrated falls over time in both adults and children, including those in high-risk areas

CDC: Centers for Disease Control and Prevention; DHS: Demographic Health Survey; NA: not applicable; NGO: nongovernmental organization.

^a^ Demographic Health Surveys, *Encuestas Nacionales de Salud* (National Health Surveys), the Health Survey for England, National Health and Nutrition Examination Surveys and national FINRISK studies are all health examination surveys.

**Table 3:** Examples of the influence of health examination surveys on health-care policy-making, Chile, England, Finland, Italy, Malta, Mexico, Peru and the United States, 1994–2020

Health topic and country	Stage at which health examination surveys influenced policy-making^a^		
	Setting the agenda	Policy formation	Policy evaluation
**Hypertension**			
Chile	Underdiagnosis and inadequate detection and treatment of hypertension identified in *Encuesta Nacional de Salud* surveys^32^	Antihypertensive drugs became free in 2014 (although *Encuesta Nacional de Salud* data provided a possible justification for this development, there was no direct link)	Reduction in salt intake; reductions in undiagnosed and untreated hypertension; and improvements in blood pressure control
England, United Kingdom	Underdiagnosis and inadequate detection and treatment of hypertension identified in Health Surveys for England^33^	General practitioner contracts were changed to incentivize blood pressure control	Reductions in undiagnosed and untreated hypertension; and improvements in blood pressure control
Peru	Underdiagnosis and inadequate detection and treatment of hypertension identified in *Encuesta Nacional de Salud* surveys^34^	NA	Reductions in undiagnosed and untreated hypertension; and improvements in blood pressure control
Mexico	In 2016, a health examination survey found that 40% of adults with probable hypertension were undiagnosed^35^	NA	NA
Italy	Between 2008 and 2012, CUORE Project health examination surveys found that 39% of men and 34% of women were unaware they had raised blood pressure^36^	NA	The 2018–2019 CUORE Project health examination survey provided evidence of improvements in awareness of raised blood pressure and in the control of hypertension^37^
**Diabetes**			
England, United Kingdom	Underdiagnosis of diabetes observed in 2009 Health Survey for England^38^	NA	NA
Finland	NA	FINRISK data used to validate the FINDRISC tool for identifying high diabetes risk^39^	NA
Italy	Between 2008 and 2012, CUORE Project health examination surveys found that 39% of men and 29% of women were unaware of probable diabetes^36^	NA	NA
Malta	The prevalence of diabetes in adults observed in the 2014–2015 health examination survey was 10.3%, with 4% of cases newly identified; the prevalence in a 2015 health interview survey was 8.5%^40^	NA	NA
Peru	Objective anthropometric data from health examination surveys were used to predict the likely numbers of cases of diabetes^41^	Weaknesses in diabetes screening programmes were identified by comparing body mass indices derived from measurements with responses to questions about screening for diabetes^42^	NA
**Hypercholesterolaemia**			
Italy	Between 2008 and 2012, CUORE Project health examination surveys found that 38% of men and 42% of women were unaware of probable hypercholesterolaemia^36^	NA	NA
**Cardiovascular risk**			
Chile	NA	Health examination survey data on the prevalence of chronic multimorbidity linked to national hospital data on discharges and deaths were used to assess the predictive validity of a new multimorbidity stratification that is now being introduced into primary care at the national level (P. Margozzini, personal communication)	NA
Finland	NA	Data from national FINRISK studies linked to national health-care data sets were used to create the FINRISK calculator for identifying individuals at a high risk of cardiovascular disease and the CAIDE risk score for predicting the subsequent development of dementia^43^^,^^44^	NA
United States	Accelerometer data from 2003 to 2006 National Health and Nutrition Examination Surveys linked to data on deaths showed that the length of physical activity bouts was not associated with the mortality benefits of accumulated moderate-to-vigorous physical activity^45^	2018 physical activity guidelines differed from 2008 guidance in dropping the requirement for aerobic activity to be performed in episodes of at least 10 minutes^46^	NA
**Chronic kidney disease**			
England, United Kingdom^47^	In 2009–2010, 3% of adults aged ≥ 75 years reported having chronic kidney disease in the Health Survey for England interview component but, according to the Health Survey for England measurements, 32% of adults had stage 3 to 5 chronic kidney disease	NA	NA
Mexico	NA	Modelling based on Chilean health examination survey data was used by the Mexican health ministry to persuade the country’s treasury to expand the range of treatments covered for end-stage chronic kidney disease (M Walbaum, personal communication)	NA
**Thyroid disease**			
Chile^48^	The 2009–2010 *Encuesta Nacional de Salud* found that the median thyroid-stimulating hormone level in the population was high, which prompted research into local causes	This finding resulted in the development of normal values for thyroid-stimulating hormone serum levels in the country and influenced Chilean hypothyroidism clinical guidelines	NA

NA: not applicable.

^a^ Demographic Health Surveys, *Encuestas Nacionales de Salud* (National Health Surveys), the Health Survey for England, National Health and Nutrition Examination Surveys and national FINRISK studies are all health examination surveys.

Data from health examination surveys can also be used to generate local hypotheses on the association between the population’s exposure to risk factors and chronic disease, to predict the future disease burden and to influence clinical and lifestyle guideline development (Table 3).

## Health policy

Data from health examination surveys can also support public health decision-making, including deciding on where policies should be targeted.

### Monitoring and establishing norms

Repeated health examination measures can be used to monitor changes in the health status of the population over time, thereby making it possible to determine whether implemented policies are having the desired effects (Table 2 and Table 3).

Even where health examination survey measurements are not used directly, they can contribute to improving other survey data. For example, Public Health England used survey data on height and weight to correct self-reported anthropometric data from the Active People Survey in England and to create an adult excess weight indicator for the Public Health Outcomes Framework.^49^ The Active People Survey included 1000 telephone participants from each local government area; that level of coverage would be far too expensive for an health examination survey.

Data from health examination surveys have also been used to determine population norms and reference values for diagnostic and laboratory tests (e.g. blood tests for thyroid hormones, liver enzymes and vitamins and urine tests for iodine excretion). Examples are listed in Table 2 and Table 3.

### Health spending

Health examination surveys can help improve the transparency and accountability of government health spending. Countries without health examination survey data on basic health indicators often base policy decisions on weak data or on imprecise estimates borrowed from other countries. Moreover, a lack of data makes it almost impossible to evaluate and justify budgets. Among the different branches of government, the health sector has always had the greatest difficulty in demonstrating the social impact of interventions and the efficiency of investments. This lack of evidence may be one reason why some countries have underfinanced health budgets. Serial cross-sectional health examination surveys can help assess trends in health indicators and the impact of policy. Without this information, health spending may lack transparency and accountability, which could affect the population’s confidence in the health authority.

## Data quality

As the use of health informatics and artificial intelligence to analyse health-care records and administrative health data is increasing, decision-makers could ask, why not replace health examination surveys with these or other data sources? However, the data obtained by health providers are usually captured using non-standardized formats: for example, different definitions of risk factor exposure or disease may be used and not everyone may be asked the same questions, as is done in surveys. Moreover, people may be receiving treatment simultaneously from different providers. In the noncommunicable disease era in particular, an individual’s journey across health and disease states is complex and difficult to register.

Health provider data usually underestimate noncommunicable disease morbidity because they exclude people who are: (i) asymptomatic; (ii) at risk of a disease but unaware of their status; (iii) symptomatic but have not sought help; (iv) have been diagnosed with a disease but have not attended regular check-ups; or (v) have died without being diagnosed. Table 3 shows examples of where health examination survey data has been used: (i) to provide accurate estimates of disease prevalence at the population level; (ii) to guide the diagnosis, treatment and control of hypertension, diabetes and hypercholesterolaemia; and (iii) to assist in the formulation and evaluation of consequent policies.

In addition, health provider-led data sources may introduce bias. The probability of being systematically recorded in a health-care database, or coded in a standardized registry, is influenced by variables that affect access to care, such as age, sex and socioeconomic and geographical characteristics – variables that are also known health determinants. Consequently, administrative data and individual health records may be biased in ways that make health inequalities appear less severe. Moreover, on occasion, provider-based financial incentives may introduce additional biases.^50^

One could argue that implementing a mandatory national notification system for chronic diseases could attenuate some of these problems. In fact, this type of surveillance has been used for communicable diseases (e.g. acute meningitis infections or chronic HIV infection) for decades with good results. However, mandatory registries are useful when disease events are infrequent but not when a disease is highly prevalent or during a pandemic. In addition, mandatory health notification systems and registries are expensive and time-consuming to maintain and validate. Although all medical specialists advocate national registries for their specific diseases, common sense tells us that doctors will not be able to complete standardized notifications for all noncommunicable diseases. Moreover, mandatory registries would not solve the problem of undiagnosed disease.

Another challenge for data collection is that health care is increasingly being provided outside traditional care structures, for example by public–private partnerships or nongovernmental organizations (NGOs) or through technology-based self-management. The result is the absence of integrated care and a lack of data interoperability. There is a need, therefore, for universal integrated surveillance of the entire population that would provide data for the design and assessment of population-wide preventive policies and for determining the coverage and performance of individual-based programmes. No matter where the population is receiving care, which health provider is involved (e.g. public, private, a NGO or culturally diverse social services) and whether individuals have adopted a self-help approach, decision-makers should be able to identify the characteristics of the people who are at risk or in need of health services. In addition, governments and health providers must be able to interpret trends in indicators (e.g. coverage of a preventive health intervention or use of a pharmacological treatment for a noncommunicable disease) and to determine whether these trends reflect a real change in people’s behaviour or risk, or are explained by changes in the places where they seek help or are registered.

### External quality control

Data from a prescription information system on dispensed drugs do not tell us which drugs have actually been taken or whether they are working effectively. In contrast, health examination survey data can provide real information about compliance and disease control. Today, the opinions of individuals are more important than ever for guiding health services. As health examination surveys draw information directly from a random sample of both users and non-users of health services, they can act as external quality controls for the entire health system. Moreover, as data are not obtained from health providers, confidence in, and the accountability of, the health authority is increased.

Recently there has been some concern that the decreasing response rates observed in health examination surveys in high-income countries may affect their external validity. However, even though response rates are decreasing, they remain higher than for mail or online surveys. Moreover, if the participants’ characteristics and the participation rate are known, study weights can be used to derive nationally representative statistics. When data are obtained instead from several health centres, data quality can be affected by a lack of standardization, interobserver variability or inter-laboratory variations in analytical processes.

### Random samples

Although health surveys may change their way of collecting information (e.g. they may become multimodal) and technological advances may improve electronic data capture, the concept of the random sample (associated with a known survey response rate) remains important. Calculations that involve denominators based on the general population are essential for guiding health policy.

Forms of data collection such as open internet surveys and random mobile phone sampling can reach millions of people at a low cost, but response rates cannot be estimated adequately. Moreover, uptake is low (response rates are generally below 30%) and these methods are prone to several selection biases associated, for example, with digital literacy, access to digital technologies or socioeconomic factors. In one project forecasting obesity in Europe,^51^ the researchers noted that data quality across the continent was highly variable and that fewer than half the countries involved possessed nationally representative, objective data. In addition, random sampling is not possible in open internet surveys because participants are all volunteers. As a result, information on event frequency is obtained only from respondents and true population prevalence rates remain unknown.

The most important characteristic of health examination surveys, in contrast, is their external validity, which enables their findings to be directly applied to the population from which their sample was drawn. When adequately powered, health examination surveys can reveal true heterogeneities in risk factor exposure and inequalities in disease burden throughout the population, which can guide the targeting of subgroups during policy development and implementation. Moreover, as health examination surveys use standardized methods, comparative analyses across countries can be performed, including analyses for Global Burden of Disease studies.

Although information technology and artificial intelligence can help develop new sampling frames and better electronic data capture systems, they cannot fix a non-random sample or non-standardized measurements of risk exposure (e.g. different definitions of a smoker) or disease prevalence (e.g. different definitions of hypertension). Consequently, non-standardized data cannot be adjusted for use in time-series analyses.

## Future directions

### Governance

To aid quality assurance and transparency, health examination surveys should be outsourced by government health authorities, and executed by independent institutions or academic groups whose unedited final reports and databases should be openly shared with the community. Some governments (e.g. in the United Kingdom) use a tendering process for a series of successive health examination surveys to promote long-term investment by universities, survey providers and laboratories. This type of health examination survey governance stimulates positive developments, including: (i) additional fund-raising by, and collaboration between, universities; (ii) continuous training of field teams and standardization of processes and procedures; (iii) the avoidance of precarious working conditions for survey staff; (iv) career development for research assistants and field staff; (v) investment in technological support for surveys; (vi) investment in the validation of new disease screening instruments (e.g. laboratory tests, examination devices and questionnaires); and (vii) opportunities for teaching and research development.

Legislative support for health examination surveys is also needed. Few countries have a track record of regular wide-ranging health examination surveys, and even fewer have secured their continuity by law. Laws should link health examination surveys directly to the iterative process used by health authorities to establish 5- or 10-year health objectives.

Using some health examination survey indicators to assess government objectives in different sectors (e.g. transport, employment, sports and physical activity, social welfare or the environment) could help promote intersectoral policy coordination and a systems-for-health approach. In addition, such linkages could help justify the financing of health examination surveys.

### Financing

Objective assessment of the impact of noncommunicable disease surveillance on health policy development, and of the avoidable costs of noncommunicable diseases could help governments justify their budget for health examination surveys. To date, however, little evidence has been disseminated about the usefulness or impact of health examination surveys or about factors influencing the effectiveness of health examination surveys. Global health initiatives should advocate that every low-resource country has its own health examination survey, and should include financing for these surveys. Initiatives like those developed for global vaccine availability during the coronavirus disease 2019 (COVID-19) pandemic could be established to combat the noncommunicable disease burden and could include: (i) international training for health examination survey project leaders; (ii) the translation of video training material for fieldworkers; and (iii) the low-cost availability of devices used in the field, laboratory materials and the point-of-care analytics used for biophysical examinations.

### Methods

Technical support and efforts to standardize surveillance methods, such as WHO’s STEPS model and DHS initiatives, are useful and should strengthen low- and middle-income countries’ ability to collect data on noncommunicable disease indicators. The availability of guidelines on using the results of health examination surveys to support the additional studies needed in the health planning cycle (Table 1) could help. Decisions on whether health examination surveys should be conducted annually or less frequently, or become a continuous programme must be made on a country-by-country basis and take financial restrictions into account.

There is space to improve, and invest in, the technologies used in the field, such as digital data capture; telephone surveys; digital disease screening (including the use of images); laboratory techniques for easier biological sampling; home-based self-sampling or self-examination; and point-of-care laboratory analytics. In the future, health examination surveys could make use of new cancer risk biomarkers, of easier and less expensive biochemical assessments of nutritional status in the general population, and of new environmental exposure biomarkers.

All health examination surveys should be designed to include consent for data linkage with vital statistics and health records. In addition, to increase analytical capacity, ministries of health should develop adequate anonymization techniques and ensure data sets are freely available. The use of standardized questions and protocols in surveys will enable comparative analyses to be conducted over time and across countries. Response rates in household surveys could be increased through social marketing and by improving community literacy, both activities that could also be supported by global initiatives.

## Conclusions

In this paper, we have added technical arguments to the debate about the importance of health examination surveys and their continued use during the post-pandemic health financing crisis, and in the context of a technological innovation boom that offers new ways of collecting and analysing individual health data. These technical arguments demonstrate that health examination surveys make an irreplaceable contribution to the local availability of primary health data that can be used in a range of further studies (e.g. burden-of-disease, cost and policy impact studies) which are essential for informing several phases of the health planning cycle (e.g. surveillance, prioritization, resource mobilization and policy development). There are solid reasons why health provider-led data sources cannot replace health examination surveys: they may underestimate morbidity and are susceptible to several types of bias. Moreover, the use of nationally representative, random samples of the general population is crucial for maintaining the external validity of any survey. In addition, health examination surveys can provide an external quality control for a country’s health system, thereby helping to ensure that the health authority’s expenditure is transparent and accountable. In the future, fieldwork for health examination surveys will probably be improved by incorporating technological innovations. Global efforts are required to help low-income countries develop the health examination surveys they need to guide policy development, implementation and assessment.
